# Leukocytoclastic Vasculitis of the Foot and Ankle: A Case Report With Over Five-Year Follow-Up

**DOI:** 10.7759/cureus.25371

**Published:** 2022-05-26

**Authors:** Ramez Sakkab, Jerry M Fabrikant

**Affiliations:** 1 Podiatric Surgery, Scripps Mercy Hospital, San Diego, USA; 2 Podiatric Surgery, Sharp Grossmont Hospital, La Mesa, USA

**Keywords:** ankle and foot, foot and ankle, cutaneous vasculitis, skin biopsy, dermatology, leukocytoclastic vasculitis, cellulitis, vasculitis

## Abstract

The estimated incidence of leukocytoclastic vasculitis of any etiology is between 15 and 30 people per million per year. Despite being a rare pathologic entity, leukocytoclastic vasculitis has many documented etiologies. Here, we report on a case of a 47-year-old man with liver cirrhosis who was admitted to our institution for diffuse palpable purpura of the distal lower extremities. Workup was largely negative for infectious and systemic causes. The patient received multiple days of intravenous antibiotics and consultations with infectious disease, dermatology, and podiatry. Skin biopsies confirmed a diagnosis of idiopathic leukocytoclastic vasculitis. A steroid taper was prescribed, and the patient had clinical resolution and healing of skin lesions. After 5.5 years after the vasculitic episode, the patient remained free of cutaneous lower extremity lesions. Medication-induced leukocytoclastic vasculitis and associations with systemic illness or malignancy were ruled out. In the lower extremities, misdiagnosis of cellulitis for noninfectious dermatologic conditions is common. Clinicians must have a wide differential and take a multidisciplinary approach to similar types of cases to reduce unnecessary antibiotic usage.

## Introduction

The estimated incidence of leukocytoclastic vasculitis (LCV) of any etiology is between 15 and 30 people per million per year [1,2]. One epidemiologic study in the United States uncovering 84 cases of histopathologically confirmed LCV between 1996 and 2010. Despite being a rare pathologic entity, LCV has many documented causes [2]. The majority appear to be IgA-mediated vasculitis and are often associated with underlying systemic disease or pharmaceuticals. In fact, recent reports estimate that nearly half of cases are associated with a systemic condition such as systemic vasculitis, autoimmune disorder(s), connective tissue disease, or malignancy [2,3].

Leukocytoclastic vasculitis itself refers to the histopathologic description of the condition as a small vessel vasculitis (SMV) [2,3]. Microscopic analysis of LCV demonstrates three characteristic findings. The first is neutrophilic infiltration around vessel walls. This includes indications of deterioration of neutrophils into nuclei remnants, termed leukocytoclasia. Second, there is general fibrin deposition about vessel walls, deemed fibrinoid necrosis. And third, there may be evidence of vessel wall and surrounding tissue injury [2]. Nevertheless, the histopathologic features must be correlated to the clinical picture.

Typical clinical patterns are cutaneous in nature. These dermatologic findings can include petechiae, palpable or nonpalpable purpura, bullous-hemorrhagic purpura, confluent purpura, urticarial wheals, and deep-skin ulcers and nodules [2,4]. In the setting of drug-induced LCV, these clinical manifestations are often skin-limited and their onset is tied to drug initiation. In both drug-induced and infectious etiologies, vasculitis onset is usually 7-10 days after drug intake or infectious provocation [2,4,5].

Here, we report on a case of a 47-year-old man with a history of liver cirrhosis secondary to alcohol use disorder. He presented with bilateral lower extremity ulcers caused by LCV, initially diagnosed as a skin and soft tissue infection (SSTI).

## Case presentation

**Figure 1:**
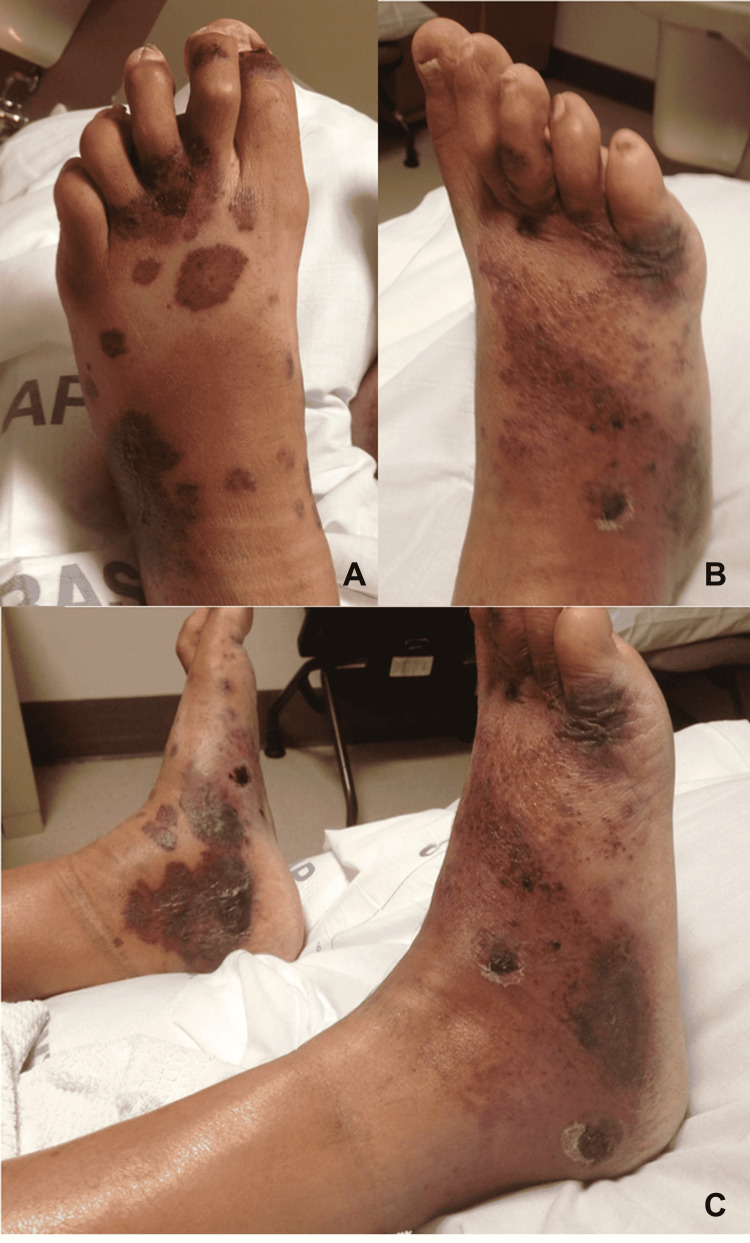
Clinical images of the patient two days into admission demonstrating bilateral cutaneous foot (A, B) and ankle (C) involvement.

A 47-year-old man was admitted to our institution from the emergency department (ED) for bilateral lower extremity wounds with surrounding edema and discoloration (Figures 1A-1C). The patient reported sudden redness and blister formation about his feet and ankles for a one-week duration. The patient was placed on broad-spectrum intravenous (IV) antibiotics (ampicillin-sulbactam) for presumed nonpurulent cellulitis. The patient had a history of dyslipidemia, hypertension, hypothyroidism, and liver cirrhosis. The patient’s liver disease was characterized as compensated, and Hepatitis C was excluded. Furthermore, vaculitidies associated with hepatitis appeared less likely as the patient had low complement proteins and normal immunoglobulin levels. The patient also had a history negative for any dermatitides. The patient had a liver biopsy at a prior separate admission which confirmed cirrhosis attributable to alcohol use disorder. The patient’s active medications prior to admission included: atorvastatin, bisacodyl, carvedilol, hydralazine, levothyroxine, ibuprofen, and lisinopril. He had been on these medications for at least one and a half years with the exception of ibuprofen. The patient’s vital signs were stable on presentation and throughout hospitalization. Physical dermatologic exam noted both feet and ankles with enlarged purple to black lesions, some raised and some flat, with bullae formation on the right trimalleolar area but none on the left. The erythema and discoloration were not blanchable. The lesions were dorsal, medial, and lateral with sparing of the plantar feet. Of note, the patient had palpable dorsalis pedis and posterior tibial pulses.

The patient was found to be anemic, with normal mean corpuscular volume, B12, and folate levels. The anemia was thus attributed to chronic alcohol use and dehydration. Other laboratory results, seen in Table 1, were within normal limits per our institution’s reference standards. Urinalysis and two blood cultures were negative. Infectious disease was consulted, prompting systemic viral and rheumatologic workup. Testing for bacterial pathogens, including staphylococcal, streptococcal, disseminated gonococcal, and atypical infections such as rodent-borne illnesses like Bartonella and Leptospira were negative. The following infectious, rheumatologic, and dermatologic panels were ordered by consulting physicians, and all negative: rheumatoid factor, cyclic citrullinated peptide, antinuclear antibody (ANA), antineutrophil cytoplasmic antibodies (ANCA), Hepatitis C and B, human immunodeficiency virus, rapid plasma reagin, cryoglobulins, Smith antigen, ribonuclear protein (RNP), Sjogren anti-SSA and SSB, and HPA platelet antibodies. Underlying malignancy was also ruled out based on patient history and the aforementioned findings.

**Table 1:** Assorted laboratory analyses on admission.

Pertinent Laboratory Markers	Value
White blood cell count (10^3^/𝜇L)	7.0
Neutrophils (%)	49.1
Hemoglobin (g/dL)	10.6
Hematocrit (L/L)	31.1
Platelets (10^3^/𝜇L)	111
Prothrombin Time (seconds)	42
Partial Thromboplastin Time (seconds)	71
Albumin, serum (g/dL)	3.4
Total protein (g/dL)	6.1
Glucose, random (mg/dL)	113
Sodium (mmol/L)	138
Potassium (mmol/L)	4.0
Chloride (mmol/L)	102
Carbon dioxide (mmol/L)	23
Anion Gap (mEq/L)	11
Blood urea nitrogen (mg/dL)	9
Alkaline Phosphatase (IU/L)	103
Aspartate Aminotransferase (IU/L)	47
Alanine Aminotransferase (IU/L)	28
Gamma-Glutamyl Transferase (IU/L)	35
Total Bilirubin (𝜇mol/L)	2.1
Direct Bilirubin (mh/dL)	0.25
Indirect bilirubin (mg/dL)	1.1
Body Mass Index (kg/m^2^)	27.04

After two days of broad-spectrum antibiotics, a computed tomography (CT) of bilateral lower extremities was ordered. A CT of the abdomen and pelvis had also been ordered and noted to be negative (including masses) with the exception of findings consistent with liver cirrhosis. Podiatry was simultaneously consulted for possible debridement versus surgical intervention. CT images were negative for bony erosions, abscess formation, and/or emphysematous changes. The bullae were lanced at the bedside, noting serosanguinous exudate. A wound culture was obtained, and mixed skin flora resulted. The attending Podiatrist (AU) then conducted a 3-mm full-thickness skin biopsy of the most central region of the largest dorsal skin lesions on each foot under local anesthesia. Dermatology was concurrently consulted.

While the differential diagnosis was broad, primary differentials included drug reaction, thrombocytopenic purpura, benign pigmented purpura, and Schamberg disease. Regarding the latter, no extravasation of blood vessels or capillaries was seen elsewhere on the lower extremities. Also, no thrombocytosis was seen as previously mentioned (Table 1). Dermatopathology results returned showing extensive red cell extravasation, some subcorneal vesicles containing blood and serum, and small vessels lined by swollen endothelial cells with some containing thrombi. Most notably, focal neutrophils were seen within wall vessels with nuclear dust present. Benign pigmented purpura was easily ruled out with histological findings given the presence of nuclear debris. Because the patient had liver disease for multiple years prior to this presentation, LCV associated with liver cirrhosis appeared less likely. And despite no acute medication changes, ibuprofen was stopped resulting in no change in dermatologic condition. In conjunction with the anatomic pathology findings, the final diagnosis was idiopathic LCV. The patient was started on IV methylprednisolone and clinical improvement was noted. The patient was prescribed a steroid taper by Dermatology on discharge. The steroid regimen consisted of prednisone 60 mg for two weeks, 40 mg for two weeks, and finally 20 mg for two weeks. The patient returned to our institution intermittently over the following years. At 5.5 years after the initial admission, continued resolution of the cutaneous lesions was confirmed with mild scarring.

## Discussion

Misdiagnosis of lower extremity edema and discoloration, especially when bilateral, is a common misstep in healthcare. Weng and colleagues noted that in cases of presumed lower extremity “cellulitis,” 30.2% were misdiagnosed [6]. With 20.7% (52) of patients being admitted for cellulitis before a noninfectious dermatologic cause was determined. Of the admitted patients, 84.6% did not require hospitalization for their final diagnosis, and over 90% received unnecessary antibiotics. They estimated 195 to 515 million dollars in unnecessary healthcare expenditure for misdiagnosed lower extremity “cellulitis” [6,7].

While our patients did receive unnecessary IV antibiotics, their condition did resemble skin and soft tissue infection was given the various ulcerations. This case further emphasizes the difficulty in diagnosing cellulitis mimics in the ED setting [7]. Furthermore, a false negative could be disastrous if a patient had not been given antibiotics. The exact risk-benefit of holding antibiotics in a possible dermatologic pathology has not been determined.

Similar diagnoses are complicated by the extensive etiologies of LCV. Systemic causes include but are not limited to medications, malignancy, systemic conditions, or infection. If no cause is determined, as in our case, it is deemed an idiopathic or unclassifiable LCV [2,8]. We surmise this LCV was associated with alcohol use and liver cirrhosis as documented previously [2], but no causal link was made. Both were present for multiple years before this bout of LCV. Thus, the diagnosis of idiopathic LCV was made. The American College of Rheumatology has previously outlined clinical criteria to diagnose LCV, which are illustrated in Table 2 [8]. The present patient met four of five of these criteria. These included: being greater than 16 years of age, having palpable purpura, maculopapular exanthema, and positive histopathology. The lone absent criteria were an association with a specific medication. Nonetheless, LCV remains a diagnosis of exclusion and is based on histopathological analysis.

**Table 2:** American College of Rheumatology Criteria for diagnosis of leukocytoclastic vasculitis.

Clinical Criteria
Age >16 years at the time of disease onset
medication use and its correlation with disease onset
palpable purpura
maculopapular exanthema
histopathological picture encompassing arterioles and venules with evidence of peri/extravascular granulocytes.
3+ of 5 criteria has a diagnostic specificity of 83.9% and sensitivity of 71%

Regarding medications, various pharmaceuticals have been associated with LCV. Recent LCV cases in the literature have been induced by direct oral anticoagulants [9]. Other cited sources of LCV are tumor necrosis factor [10] and specific antibiotics [11,12]. Antirheumatic agents, such as etanercept, have also been associated with LCV [13]. More commonly, anticoagulants have also been implicated in cases of LCV [14]. In one case, the patient had been taking warfarin for four years and the skin eruptions occurred with rechallenge of warfarin [14]. Here, only a rechallenge of ibuprofen was conducted. Rechallenge of other patient medications could easily be indicated but was avoided in this case. Furthermore, although heparin was started for prophylaxis on admission, the patient was not taking any anticoagulation agents prior to presentation. Ibuprofen was stopped to confirm it had not contributed to LCV and began again without incident. The lack of clinical response prompted further workup and consultation.

Malignancy has been closely associated with cutaneous LCV [14]. Immunological workup for underlying malignancy in our patient was negative. This workup included normal cryoglobulins, anticardiolipin antibodies, total serum hemolytic complement, and complement proteins in addition to laboratory results as seen in Table 2. Patients seen in prior studies had positive autoantibodies, most commonly ANA and Jo-1, in LCV associated with malignancy [14]. Unfortunately, direct immunofluorescence study was not obtained. Furthermore, dermatomyositis with LCV is a common finding when malignancy is involved [2,14]. In our patient, muscle involvement was not appreciated. Previous reports noted muscle involvement in all LCV cases with concurrent malignancy [14,15].

A seminal work on LCV was that of Ekenstam and Callen in 1984 [15]. They found that 42 of their 82 patients (51.2%) had underlying systemic disease, and a relationship with LCV was presumed. Despite frequent systemic findings, clear association with systemic disease was not proven in our case. Leading to a diagnosis of idiopathic LCV. Moreover, it appeared to be acute LCV, defined as resolving within six months [14,15]. Given the lack of history and continued resolution of symptoms after five years. Ekenstam and Callen noted LCV was acute, chronic, and relapsing in 56%, 28%, and 16% of cases, respectively. It does seem that the majority of patients respond to treatment, with approximately 10% of cases resulting in recurrence of lesions [16]. Our patient not only responded to therapy but also did not have a reoccurrence over 5.5 years.

A recent systematic review was undertaken reviewing LCV secondary to ulcerative colitis (UC) [17]. Although not present in our patient, UC is known to produce LCV as a rare reactive skin manifestation [17]. In Pantic and colleagues' systematic review, 22 cases were reviewed, with the lower extremities most commonly involved (73%). While negative in our case, ANCA was positive in 41% of UC patients. Overall, LCV was misdiagnosed in 22.7% of cases. Interestingly, 82% of these patients' rash resolved without scarring, which was not the case in our patient presenting without UC.

Moreover, previous works have found that systemic causes account for the majority of LCV cases [2,18] However, in a relatively large case series of 93 patients, idiopathic cases accounted for 44.1%. And extracutaneous involvement was not discovered in 60.2% of cases [18]. Extracutaneous involvement, despite extensive workup, was negative in our patient. This further classifies the case as a single-organ vasculitis. Although idiopathic causes of LCV may be overreported [1,2], no other vasculitis occurred throughout the five plus years of patient follow up at our institution. He also did not suffer from a new systemic diagnosis over that time. In recent years, LCV has also been found to have clinical correlation with coronavirus disease 2019 (COVID-19), both the primary viral illness and its vaccines [19]. While our patient presented in 2016, other viral agents were considered.

Outside of vasculitis, another pertinent differential was pyoderma gangrenosum (PG). PG is a neutrophilic dermatosis caused by an autoinflammatory response. PG is associated with similar appearing skin lesions with peripheral erythema but in the case of PG also containing undermined borders [20]. PG and LCV both have a wide range of differentials and require a histopathologic diagnosis. However, debridement of PG ulcerations causes progression and worsening. It is important to consider PG as a differential to avoid debridement and thus greater skin and soft tissue destruction.

## Conclusions

Multidisciplinary discussion of the above case led to more prompt procedures, diagnostic conclusions, antibiotic avoidance, and treatment regimen. LCV has many etiologies, and drug-induced LCV should always be ruled out. Despite a majority of cutaneous LCV cases being associated with malignancy or medications, both were meticulously ruled out in our patient. A prompt skin biopsy as early in the clinical course as possible is key. Our patient had complete resolution of symptoms and healing of ulcers with a 6-week tapered course of prednisone. Interestingly, no causal agent or associated condition was determined. To the authors knowledge, this report demonstrates the longest follow up in the literature of lower extremity LCV. And his lower extremity wounds remained healed at 5.5 years of follow up.
